# Nonlinear relationship between viral load and TCT in single/multiple HPV52 infection

**DOI:** 10.1186/s12985-024-02356-4

**Published:** 2024-04-23

**Authors:** Bingbing Ma, Jie Zhou, Weijuan Zhou, Zhanzhong Ma, Juan Chen, Hongbo Hu

**Affiliations:** 1 Department of Gynecology, Yuebei People’s Hospital affiliated to Shantou University Medical College, Shaoguan, China; 2 Reproductive Medicine Center, Yuebei People’s Hospital Affiliated to Shantou University Medical College, Shaoguan, China

**Keywords:** HPV viral load, Single HPV infection, Multiple HPV infections, TCT

## Abstract

**Purpose:**

To determine the correlation between HPV (human papillomavirus) 52 viral load, multiple infections and ThinPrep cytology test (TCT), to inform clinical management of HPV52-positive women after cervical cancer screening.

**Methods:**

A total of 1,882 female patients who had positive quantitative HPV tests at Yuebei People's Hospital from January 2020 to December 2022, of whom 533 tested positive for HPV52. We excluded patients who combined HPV16 and/or HPV 18 positivity and whom HPV52 viral load could not be calculated. The final enrollment was 488 patients, including 400 NILM, 48 ASC-US, 28 LSIL and 12 HSIL. The HPV test is a quantitative multiplexed fluorescent PCR assay that provides both HPV genotyping and viral load.

**Results:**

In our study, there were differences in the median distribution of viral loads among various cytological class categories. The risk of TCT results (LSIL or worse) was increased with the increase of HPV52 viral load, for every LOG unit increase in HPV52 viral load, the risk increased by 26.6%. More importantly, we found a nonlinear relationship between HPV52 viral load and TCT results (LSIL or worse) in both single and multiple infections. When the viral load reaches a threshold, the risk of abnormal cytological results increases significantly.

**Conclusion:**

HPV52 viral load is an independent risk factor for TCT results (LSIL or worse). The relationship between HPV52 viral load and TCT results (LSIL or worse) is not linear. Viral load may be used as a triage indicator for HPV52-positive patients, thus improving the post-screening clinical management of HPV52-positive women.

**Supplementary Information:**

The online version contains supplementary material available at 10.1186/s12985-024-02356-4.

## Background

Cervical cancer arises from the primary oncogenic types of human papillomavirus, ranking as the fourth most prevalent malignant tumor among women worldwide [1, 2]. About 20 genotypes of HPV viruses infecting the reproductive system are associated with cancer development. Of these, HPV types 16, 18, 31, and 52 fall under the classification of "high-risk" types [2]. Prolonged exposure to high-risk human papillomavirus (HR-HPV) can result in the development of cervical intraepithelial neoplasia and even cervical cancer [3]. The incidence and mortality of cervical cancer have been increasing annually, the mortality burden associated with cervical cancer remains heavy, early screening and treatment of cervical cancer are very important and necessary [1, 4–6].

HPV tests offer a more precise method for detecting advanced lesions in cervical cancer screening, yet their specificity is restricted. It is imperative to discover a diagnostic marker that possesses both sensitivity and specificity. Apart from the type of HPV, there are other factors associated with HPV such as the viral load [7, 8]and the variety of types [9–11]. The majority of research has established a positive association between HPV viral load and cervical lesions [12–14], indicating that high HPV viral load elevates the probability of high-grade cervical lesions. Additionally, Wang, W’s study [15] highlighted the correlation between HPV viral load and the extent of cervical lesion, emphasizing the necessity of incorporating it into a distinct genotype. Numerous studies have been conducted on HPV types 16 and 18, serving as a valuable resource for guiding clinical post-screening management [16], while fewer studies have been conducted on other high-risk genotypes.

HPV-16, 18, 52, 31, and 58 [17, 18] are the prevailing high-risk HPV types in Asia. Many epidemiologic data indicate that HPV 52 presently stands as the predominant form of infection among women in southern China [19, 20]. According to the ASCCP guidelines, patients positive for the high-pathogenicity virus subtypes 16/18 are recommended to undergo further colposcopy and pathological biopsy, however, patients who test positive for other (non-16/18) high-risk HPV types should undergo combined cytological testing to inform the decision-making process regarding their referral. In order to better guide this population, we conducted a retrospective analysis of the data of HPV52 infected females in our hospital during the 3-year period from 2020 to 2022, to analyze the correlation between multiple infections, HPV52 viral load, and TCT results, to find out whether the HPV52 viral load holds predictive and triage potential in cervical cancer screening.

The aim of this article is to summarize the stratification effect of genotypes, multiple infections, especially viral load in cervical screening. Our goal is to provide evidence that can inform post-genotype-specific screening management strategies.

## Materials and methods

### Study participants

This retrospective study included women who tested 52 positive for HPV viral load between January 2020 and December 2022 at Yuebei People's Hospital. The Ethics Committee of Yuebei People's Hospital gave the go-ahead to the research, which was done in line with the Declaration of Helsinki and its associated rules and regulations. All patients signed a written informed consent form before the examination. As a retrospective study, the clinical data of patients were obtained from the HIS system of Hospital. The data collection process was confidential, and sensitive information of the patients was anonymized during the collection and analysis process, and there were no costs or additional risks to patients.

Inclusion criteria were (1) HPV DNA52 positive patients (2) Patient had TCT results for the same period of time. Exclusion criteria were (1) patients with high-risk HPV16 and/or HPV 18 co-infection. (2) Incomplete information.

**Fig. 1:**
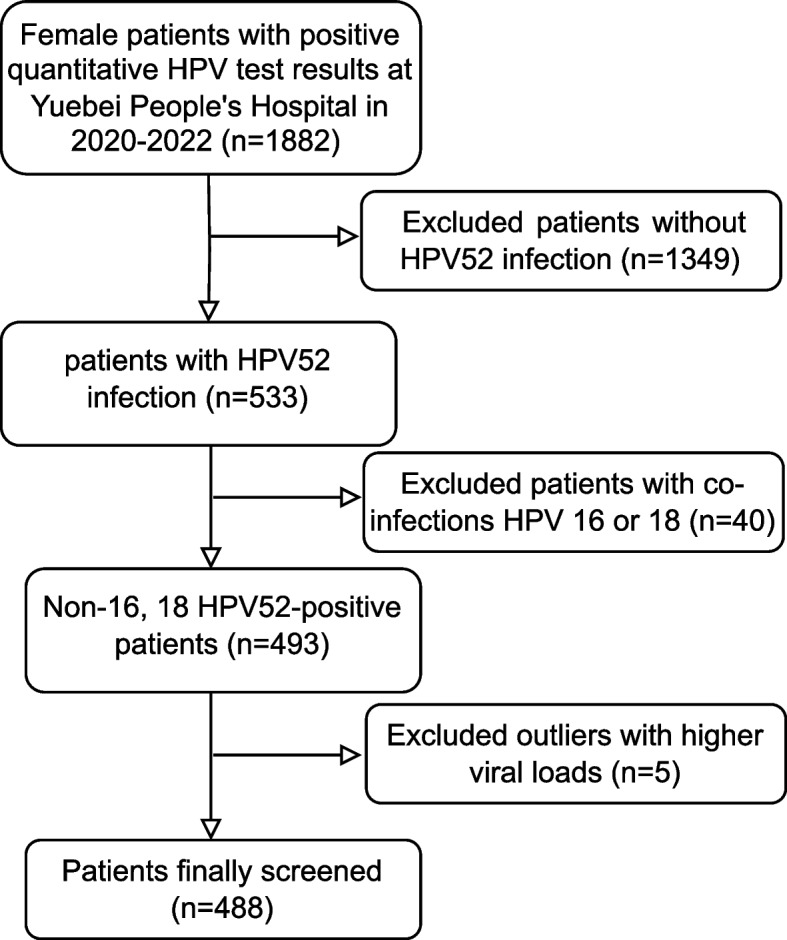
This flow chart that screening the participants in this study

Between 2020 and 2022, a total of 1882 female patients who underwent HPV quantitative testing at Yuebei People's Hospital tested positive, with 533 of them testing positive for HPV52. We omitted 40 patients who tested co-positive with HPV16/18, and we removed 5 patients whom HPV52 viral load could not be calculated. In the end, a total of 488 patients were incorporated (Fig. 1).

### Data collection and grouping

Clinical data such as patient's age, HPV52 viral load, viral load of other types (without this data it is a single infection), and contemporaneous TCT results were obtained from clinical record through Yuebei People's Hospital's HIS system.

### HPV Genotyping and Viral Load

The HPV genotyping test kits applied by Yuebei People’s Hospital was provided by 21 HPV genotyping Kit (Shuoshi Biotechnology, Ltd., China, Jiangsu), The test kit was approved by the National Medical Products Administration (NMPA, No. 20153400364). The kit adopts multiplex fluorescent PCR quantification technology, which can rapidly and accurately distinguish 21 HPV genotypes in the test specimen, including 13 HR-HPV16, 18, 31, 33, 35, 39, 45, 51, 52, 56, 58, 59, 68, intermediate-risk HPV26, 53, 66, 73, 82, and 3 low-risk HPV6, 11, 81, and also simultaneously quantify the viral loads of the 21 HPV subtypes in a standardised manner.

This Kit targets the L1 region of the human papillomavirus genome, using PCR primers and corresponding TaqMan probes (oligonucleotides with a 5' reporter group and a 3' quencher group) for detection, with FAM, HEX and ROX marking the respective subtypes, and running 8 reactions simultaneously for each sample. The preparations for reactions A, B, C, D, E, F and G are used for the simultaneous detection and differentiation of 21 HPV genotypes. In reaction H, t the single-copy gene TOP3 encoding DNA topoisomerase III is amplified as a control to determine the viral copy number in a given sample. The sensitivity of the assay is 20copies /reaction.

Sampling and extract the sample DNA according to kit requirements. The total reaction volume is set to 20 μL, consisting of 2 μL DNA sample, 10 μL nucleic acid amplification reaction liquid, and 8 μL reaction liquid (including specific primers and probes). After mixing all the components, the reaction tube is placed in a fluorescence PCR amplifier for amplification detection. The reaction conditions 77 were as follows: treatment of UNG enzyme at 50 ℃ for 5 min, pre-denaturation at 95 ℃ for 10 min, denaturation at 94 ℃ 78 for 10 s, annealing, extension and fluorescence detection at 58 ℃ for 40 s, cycling 45 times, and storage at 4 ℃.

Finally, subtypes were analyzed according to the HPV subtyping probe fluorescent marker table. The obtained CT values were substituted into the HPV nucleic acid typing quantitative analysis software v1.0 (Shuoshi Biotechnology, Ltd., China, Jiangsu) for transformation. Absolute quantification was mainly performed by establishing a five-point standard curve of HPV and cell log phase. The standard curve was Y = -3.34656 (log10X) + 38.51644. the converted viral load unit was the number of cells (pcs).

### ThinPrep cytology test(TCT)

Cervical ThinPrep cytology test was interpreted by two senior specialists in the pathology laboratory. The diagnostic results of the TCT were classified according to the Bethesda system by the International Cancer Society (2014), Results were categorized into five classes: negative for intraepithelial lesion or malignancy (NILM), atypical squamous cells of undetermined significance (ASCUS), atypical squamous cells–cannot exclude high-grade squamous intraepithelial lesion (ASC-H), low-grade squamous intraepithelial lesion (LSIL), high-grade squamous intraepithelial lesion(HSIL) and squamous cell carcinoma (SCC). There are 6 cases of ASC-H,5 cases of HSIL and 1 case of SCC. Since there were too few cases and those results need to be referred for the next step of colposcopic biopsy, so we merged them into the TCT results of HSIL. To further validate the relationship between HPV52 viral load and TCT, we divided four categories into two groups for comparison. Given the specificity of ASCUS, it can either indicate a benign lesion or signify a potentially malignant alteration associated with active proliferation. So we grouped TCT subgroups were stratified into groups: one is TCT results of ASCUS or better; the other is TCT results of LSIL or worse.

### Statistical analysis

SPSS 24.0 (IBM, Armonk, NY, USA) and R language software was used for data processing and statistical analysis. TCT results were used as grouping variables to describe the study population. Age and log-transform-processed HPV52 viral loads were normally distributed continuous variables, quantitative data were expressed as mean ± standard deviation. The type of HPV infection was a categorical variable and was expressed as a percentage. Differences between groups were evaluated using the ANOVA and Kruskal Wallis rank sum tests. *p*-values < 0.05 were considered statistically significant.

The HPV52 viral load data showed a skewed distribution among the participants, therefore, we processed the HPV52 viral load by log transformation. The results of data analysis were divided into 3 models: model 1 (no covariates were adjusted); model 2(adjusted age); and model 3 (adjusted age and infection types). Confidence intervals (CI, 95%) and odds ratios (OR) were used to screen the adjusted covariates included in the models, while univariate analysis of p values did not yield the same results. A generalized additive model was used to adjust for all covariates, assess nonlinear relationships, and plot smooth curves. In the presence of nonlinearity, we employed a recursive algorithm to determine the inflection point. Then a segmented linear model was constructed based on both sides of the inflection point. The *p*-value of the log-likelihood ratio test was used to construct the best-fit model. If the *p*-value was ≤ 0.05, the correlation between viral load and TCT was nonlinear, otherwise it was linear. In the single infection curve, we got two inflection points K1 (8.95); K2 (11.35) (Table 5). In the multiple infection curve, there is only one inflection point K3 (12.095) (Table 6). Multiple infections include both (non-16/18) high-risk and low-risk HPV types.

## Results

### Baseline characteristics of selected participants

A total of 488 non-16/18 HPV52 positive women were included in this study. Among them, 400 exhibited normal cytologic findings, 48 tested positive for ASC-US, 28 tested positive for LSIL, and 12 tested positive for HSIL (Table 1), there were 6 positive for ASCH and 1 positive for SCC included into HSIL. The age distribution showed no notable disparity among the various TCT groups (*P* = 0.066). HPV 52 viral load was higher in the other TCT groups (ASC-US, LSIL, HSIL) compared to the NILM group, and the difference was statistically significant (*P* < 0.001). Additionally, the percentage of mono-infections was higher than that of multi-infections among all TCT groups, with a statistically significant disparity (*P* = 0.017).

**Table 1:** Baseline characteristics of selected participants

TCT	NILM	ASC-US	LSIL	HSIL	*P*-value	*P*-value*
	**(*N* = 400)**	**(*N* = 48)**	**(*N* = 28)**	**(*N* = 12)**		
AGE(mean ± SD)	40.615 ± 11.191	43.271 ± 11.169	39.857 ± 11.639	47.917 ± 12.442	0.066	0.104
HPV52LOAD Log (mean ± SD)	8.244 ± 2.710	9.471 ± 3.052	9.969 ± 3.308	10.645 ± 2.346	< 0.001	< 0.001
TYPE					0.017	-
Single(N;%)	309 (77.250%)	30 (62.500%)	16 (57.143%)	10 (83.333%)		
multiple(N;%)	91 (22.750%)	18 (37.500%)	12 (42.857%)	2 (16.667%)		

*P*-value*: The Kruskal Wallis rank sum test yielded a *P*-value for continuous variables, while Fisher's exact probability test was employed for count variables with a theoretical number < 10

### Baseline Characteristics and TCT results (LSIL or worse)

The TCT results were divided into ASCUS or better group and LSIL or worse group for analysis. Age was analyzed in low, middle and high three subgroups (Table S2). With increasing age, the risk of TCT results (LSIL or worse) also increased, although it did not reach statistical significance (all *P* > 0.05). In the comparison of infection types, the risk of TCT results (LSIL or worse) rose by 67.5% in the multiple infection group when comparing infection types to the single infection group, but the statistical significance was not achieved (*P* = 0.139). For HPV52 viral load, with an increase in HPV52 viral load, there was a significant rise of 25.5% (OR: 1.255, 95% CI: 1.114–1.415) in the risk of TCT results (LSIL or worse), indicating a highly significant statistical significance(*P* < 0.001) (Table 2).

**Table 2:** Relationship of baseline characteristics and TCT results (LSIL or worse)

Exposure	TCT
	**OR, 95% CI, *p*-value**
AGE	1.011 (0.982, 1.040) 0.460
AGE tertile	
Low	1.0
Middle	1.182 (0.529, 2.642) 0.682
High	1.167 (0.522, 2.606) 0.706
TYPE	
Single	1.0
Multiple	1.675 (0.844, 3.321) 0.139
HPV52LOAD Log	1.255 (1.114, 1.415) < 0.001

### HPV52 Viral load and TCT results (LSIL or worse)

In order to establish the relationship between HPV52 Viral load and TCT results (LSIL or worse), three models were examined to analyze the pattern of effect values in Table 3 (OR and 95% CI).In model 1 (unadjusted model), The risk of TCT results (LSIL or worse) showed a significant increase of 25.5% (OR: 1.225, 95% CI: 1.114–1.415) with each loge unit increase in HPV52 viral load (*P* < 0.001).After conducting a more thorough examination, we divided the viral load into three distinct subcategories: low, medium, and high (Table S1). Compared with the low viral load group (5.451), the risk of medium viral load (8.338) was not significantly increased, while the risk of high viral load (11.739) was significantly increased by 2.3 fold (OR: 3.300, 95% CI: 1.436–7.585), indicating statistical significance (*P* = 0.005). Both model 2 (adjusted age) and model 3 (adjusted age, infection type) revealed a significant correlation between increasing HPV52 viral load and the risk of TCT results (LSIL or worse), with 26.9% and 26.6% increase respectively (*P* < 0.001). When analyzing the three categories of viral load, there was also a statistically significant (*P* = 0.003/ *P* = 0.002) increase in risk in the high viral load group by a factor of 2.557 (medol2), 2.669 (model3) respectively. This indicates that TCT results (LSIL or worse) can be attributed to HPV52 viral load as a distinct risk factor.

**Table 3:** Linear relation of HPV52 Viral load and TCT results (LSIL or worse) by the weighted binary logistic regression model

Exposure	Model 1	Model 2	Model 3
	**OR, 95% CI, *p*-value**	**OR, 95% CI, *p*-value**	**OR, 95% CI, *p*-value**
HPV52LOAD Log	1.255 (1.114, 1.415) < 0.001	1.269 (1.124, 1.432) < 0.001	1.266 (1.122, 1.428) < 0.001
HPV52LOAD Log tertile			
Low	1.0	1.0	1.0
Middle	1.000 (0.366, 2.732) 1.000	1.025 (0.374, 2.808) 0.961	1.078 (0.392, 2.966) 0.88
High	3.300 (1.436, 7.585) 0.005	3.557 (1.527, 8.284) 0.003	3.669 (1.567, 8.589) 0.002

Model 1: no covariates were adjusted

Model 2: age was adjusted

Model 3: all covariates presented in Table 1(age; type) were adjusted

We analyzed the population by dividing it into single and multiple infections. Our findings revealed a significant correlation (*P* < 0.001) between an increase in load and an 55.6% higher risk of a TCT result of LSIL and above in the single-infection population. The risk of TCT results (LSIL or worse) experienced a 6.4% decrease in the population with multiple infections, albeit not attaining statistical significance (*P* = 0.498). The results of the interaction test indicated a significant disparity in this effect between single and multiple infected participants (*P* < 0.001) (Table 4). When age was analyzed by subgroups (Table S2), despite the higher age group experiencing an 46% increase in the risk of cytology abnormality, the interaction effect analysis did not indicate a significant distinction among the low, middle, and high age subgroups (*P* = 0.235).

**Table 4:** Relationship between viral load and TCT results (LSIL or worse) in different age groups and infection types

TCT	N	OR	95%CI Low	95%CI High	*P* value	P(interaction)
AGE tertile						0.235
Low	162	1.177	0.964	1.438	0.110	
Middle	162	1.163	0.942	1.437	0.160	
High	164	1.460	1.174	1.816	< 0.001	
Total	488	1.261	1.118	1.423	< 0.001	
TYPE						< 0.001
Single	365	1.556	1.304	1.858	< 0.001	
Multiple	123	0.936	0.774	1.132	0.498	
Total	488	1.251	1.111	1.408	< 0.001	

### The Nonlinearity of HPV52 Viral load and TCT results (LSIL or worse)

**Fig. 2:**
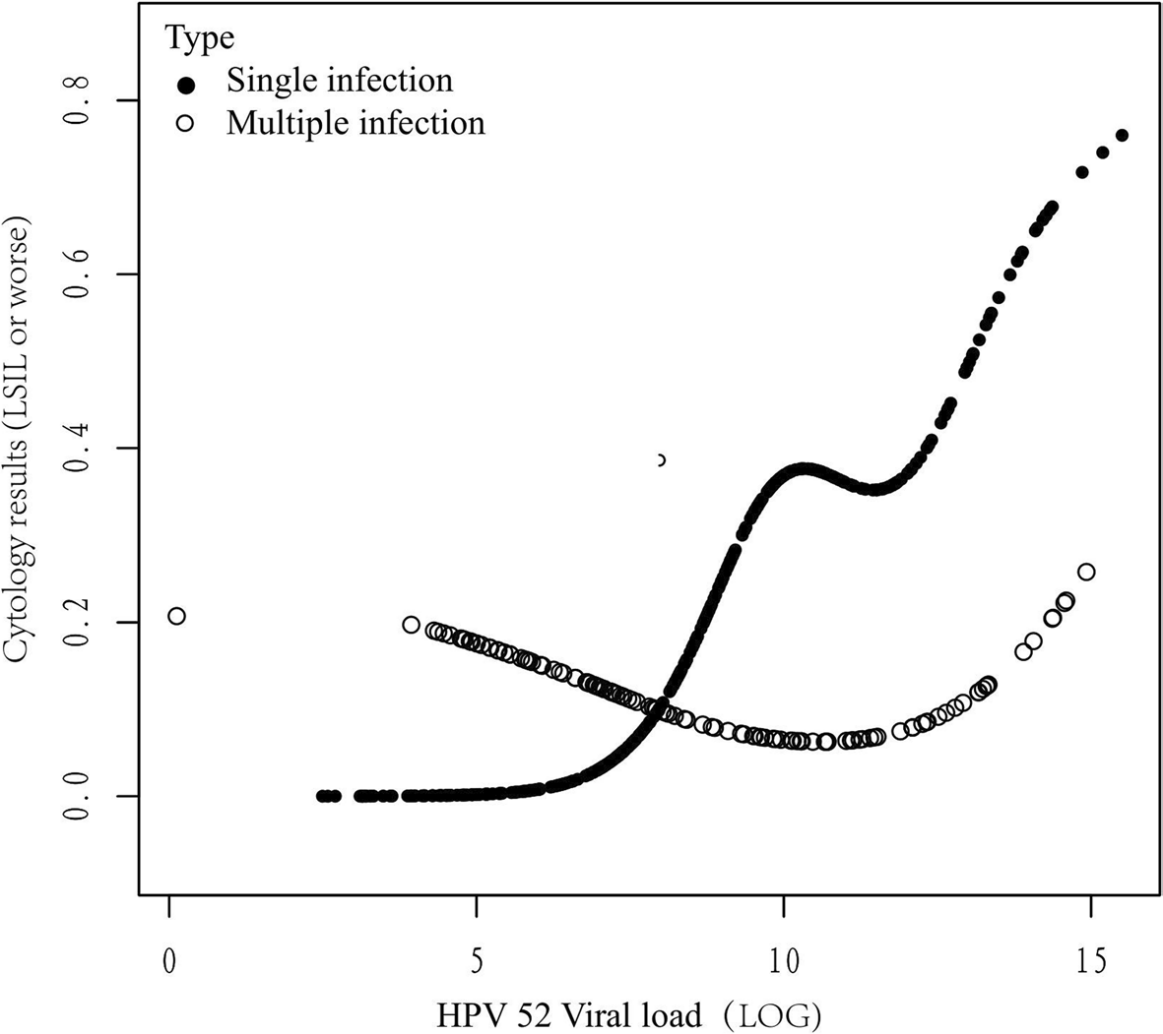
A non-linear relationship between HPV52 Viral load and TCT results (LSIL or worse) in single and multiple HPV infection

Based on model 3 (adjusted for age and type), smoothed curve fitting showed a nonlinear relationship between HPV52 viral load and TCT results (LSIL or worse) (Fig. 2). In the single-infected population, the two inflection points are 8.95 and 11.35 of loge HPV52 viral load in this curve (Table 5). Between 0–8.95, for each one unit increase in log HPV52 viral load, there was a significant 4.508-fold increase in the risk of having TCT results (LSIL or worse) (OR: 5.508 95% CI: 1.206–25.145) (*P* = 0.028). Within the range of 8.95–11.35, there was no significant 10.4% increase in the risk of TCT results (LSIL or worse) (*P* = 0.812). However, when log HPV52 viral load excess 11.35, there was once again a statistically significant 99.5% rise in the risk of TCT results being LSIL or worse (OR: 1.995 95% CI: 1.061–3.749) (*P* = 0.032).

**Table 5:** Nonlinearity addressing between HPV52 Viral load and TCT results (LSIL or worse) in single HPV infection

Outcome	Single infection
	**OR, 95% CI, *p*-value**
Fitting model using the weighted-logistic regression model	1.566 (1.312, 1.869) < 0.001
Fitting model using the weighted three-piecewise linear model	
Inflection point (K1, K2)	8.95, 11.35
≤ inflection point K1	5.508 (1.206, 25.145) 0.028
Between K1 and K2	1.104 (0.489,2.495) 0.812
> inflection point K2	1.995 (1.061, 3.749) 0.032
*P* for the log-likely ratio test	0.008

The viral load in the population with multiple infections exhibited a U-shaped curve correlation with TCT results (LSIL or worse), with an inflection point of 12.095 (Table 6). In the range of 0–12.095, for each unit increase in log HPV 52 viral load, the risk of having TCT results (LSIL or worse) was reduced by 23.2%, but this was not statistically significant (*P* = 0.053). However, once the value excess 12.095, the risk of TCT results (LSIL or worse) increased 2.025-fold, which was statistically significant (*P* = 0.028).

**Table 6:** Nonlinearity addressing between HPV52 Viral load and TCT results (LSIL or worse) in multiple HPV infection

Outcome	multiple infection
	***OR, 95% CI, p***-value
Fitting model using the weighted-logistic regression model	0.939 (0.775, 1.139) 0.523
Fitting model using the weighted two-piecewise linear model	
Inflection point (K3)	12.095
≤ inflection point	0.768 (0.588, 1.004) 0.053
> inflection point	3.025 (1.128, 8.116) 0.028
*P* for the log-likely ratio test	0.023

## Discussion

In this extensive cross-sectional investigation, we investigated the correlation between HPV52 load and TCT results (LSIL or worse), while excluding the extremely pathogenic genotypes HPV16 and HPV18, exclusively analyzing HPV52 viral load to forecast the risk of TCT results (LSIL or worse). A strong correlation was observed between viral loads and an elevated susceptibility to cytology abnormalities. Additionally, our research indicates that there was a nonlinear correlation between HPV52 viral loads and the risk of TCT results (LSIL or worse), both in single and multiple infections.

The value of particular HPV genotypes and their viral load in cervical lesions and diagnosis has been controversial [21–23]. Many studies have suggested a positive correlation between the viral load of HPV16 and the extent of cervical lesions [24, 25]. Similar findings exist for HPV18 viral load [14], however, the predictive importance of viral load in other high-risk categories remains a subject of debate [15]. We performed a retrospective analysis to investigate the correlation between HPV 52 viral load, infection types, and cytology results. The findings indicated that the TCT results (LSIL or worse) exhibited a higher HPV52 viral load. Comparison with NILM and ASCUS group, each unit increase in HPV52 viral load was associated with a 26.6% increase in viral load. Even after adjusting for external factors, the notable association was remained. HPV52 viral load poses an independent risk for abnormal cytology results. In areas where HPV52 is the primary type of HR-HPV infection, performing concurrent viral load testing during the initial screening could offer more valuable perspectives for its post-screening management [13].

We further stratified the study population into HPV52 single infection and co-infection with other (non-16/18) genotypes, we discovered that the risk of abnormal TCT was higher in HPV52 single infection, although this risk did not exhibit a significant increase in multiple infections. The increased susceptibility to disease in mono-infection compared to multi-infection could be attributed to the pathogenicity of particular genotypes, such as that HPV 16 mono-infected patients have a higher incidence of CIN2 + than patients co-infected with other HPV genotypes [26], and it is worth noting that both HPV 16 and HPV 52 belong to the α-9 genus [27]. The pathogenicity of the HPV 52 genotype also should be emphasized.

We further analyzed the correlation between HPV52 single/multiple infection combined viral load and the risk of TCT results (LSIL or worse). The viral load of HPV52 single infection and TCT results (LSIL or worse) showed a non-linear correlation, the viral load in the range of less than 8.95 and more than 11.35, with the increase of each one unit load, the risk increased, but when the viral load between 8.95–11.35 curve appeared to be a plateau. As the load increases, the risk of TCT results (LSIL or worse) ceases to rise. The strong self-replicating ability of HPV may be attributed to the fact that the low-grade lesions are in the acute phase of HPV infection. As the lesion advances, the HPV viral load no longer experiences a substantial rise owing to the relative stabilization of HPV's self-replicating capability; nevertheless, when HPV genes become integrated into the host cell's DNA, the viral load appears to increase once again [28, 29], exacerbating the disease even further.

There was also a nonlinear correlation observed in HPV52 multiple infection. Interestingly, the curve was different from the unidirectionality of the curve for single infection, but showed a bidirectional U-shaped curve, and the inflection point of this curve was 12.095. When the LOG viral load was prior to the inflection point, the risk of TCT results (LSIL or worse) did not increase, but instead exhibited a decreasing trend (OR:0.768 95% CI: 0.588–1.004); whereas, the risk of TCT abnormality increased 2.025-fold after the load exceeded 12.095. We analyzed that the appearance of the U-shaped curve might be related to inter-genotype competition or the heightened immune response induced by multiple infections [30]. Only when the viral load exceeds a certain threshold, the pathogenicity of the viral load exceeds the immunity of the organism and then causes lesions, that is, we see completely opposite pathogenic risks from both sides of the curv1e's inflection poin. Through the appearance of the inflection point of viral load, we can differentiate the risk of HPV52 multiple infection in peoples, we can categorize the population with viral load before the inflection point into a "relatively low-risk group", which needs to be closely followed up with the change of viral load; When viral load exceeds the inflection point, the risk of TCT results (LSIL or worse) increases significantly, and timely referral for colposcopy and biopsy may be recommended for efficient detection of high-grade lesions.

HPV genotype testing, which is used to screen for cervical cancer, is highly sensitive but not very specific. Through this study, we investigated the correlation between viral load and TCT results in relation to a specific genotype, HPV52, and ascertained that viral load can function as a diagnostic indicator for patients who tested positive for HPV52. The HPV52 viral load has the potential to enhance the clinical care of women who test positive for HPV52, by closely monitoring the viral load before the inflection point. This approach not only reduces the need for invasive procedures and economic burden, and it is more meaningful for population screening in less economically developed areas.

As this study only included data from a single center and excluded cases of multiple infections with HPV16/18, the participants size was relatively limited, and it may be necessary to expand the number of cases to further validate the cut-off value of viral load proposed in this study. This study only explored the relationship between viral load and thinprep cytology test results, and verified that viral load might be used as an indicator for the triage of HPV52-positive patients, and subsequent pathological information is needed to validate it, which is also our future research direction.

## Conclusions

This retrospective study showed that HPV52 viral load was an independent risk factor for abnormal cytology results. More importantly, there is a nonlinear relationship between HPV52 viral load and TCT results (LSIIL or worse). Whether in single infection or co-infection with other HPV genotypes, once the viral load reaches thresholds, the risk of abnormal cytology results significantly increases. Viral load has the potential to enhance the clinical management of HPV 52 positive women in cervical cancer screening.

### Supplementary Information

**Additional file 1: Table S1.** HPV 52 LOAD tertile.

**Additional file 2: Table S2.** AGE tertile.

## Data Availability

No datasets were generated or analysed during the current study.

## Declarations

**Ethics approval and consent to participate**

The study was conducted in accordance with the Declaration of Helsinki, and this study was obtained from the Ethics Committee of the Yuebei People’s Hospital (KY-2023–135). All patients are completely anonymous and free from any personal privacy. All methods are carried out by relevant guidelines and regulations.

**Consent for publication**

The authors agree to publication.
