# Supplementary material for: Nonlinear relationship between viral load and TCT in single/multiple HPV52 infection
Source: Virol J. 2024 Apr 23;21:90. doi: 10.1186/s12985-024-02356-4 (PMC11036758; doi:10.1186/s12985-024-02356-4)
Supplement: Supplementary file 1 — Additional file 1: Table S1. HPV 52 LOAD tertile. [file 12985_2024_2356_MOESM1_ESM.docx]

Table S1. HPV52LOAD Log tertile

| HPV52LOAD Log tertile | Low | Middle | High | P-value | P-value* |
| --- | --- | --- | --- | --- | --- |
| N | 162 | 162 | 164 |  |  |
| HPV52LOAD Log | 5.451 ± 1.236 | 8.338 ± 0.813 | 11.739 ± 1.419 | <0.001 | <0.001 |

表中结果: Mean+SD / N(%) 
P值*: 如是连续变量，用Kruskal Wallis秩和检验得出, 如计数变量有理论数<10，用Fisher精确概率检验得出.
