# Supplementary material for: Nonlinear relationship between viral load and TCT in single/multiple HPV52 infection
Source: Virol J. 2024 Apr 23;21:90. doi: 10.1186/s12985-024-02356-4 (PMC11036758; doi:10.1186/s12985-024-02356-4)
Supplement: Supplementary file 2 — Additional file 2: Table S2. AGE tertile. [file 12985_2024_2356_MOESM2_ESM.docx]

Table S2. AGE tertile

| AGE tertile | Low | Middle | High | P-value | P-value* |
| --- | --- | --- | --- | --- | --- |
| N | 162 | 162 | 164 |  |  |
| AGE | 28.44 ± 4.37 | 40.82 ± 3.63 | 53.78 ± 5.70 | <0.001 | <0.001 |

表中结果: Mean+SD / N(%) 
P值*: 如是连续变量，用Kruskal Wallis秩和检验得出, 如计数变量有理论数<10，用Fisher精确概率检验得出.
